# Hidden pathway: the role of extracellular matrix in type 2 diabetes mellitus–related sarcopenia

**DOI:** 10.3389/fendo.2025.1560396

**Published:** 2025-04-16

**Authors:** Yiping Sun, Zepeng Zhang, Yufeng Wang, Xingquan Wu, Yahui Sun, Huijuan Lou, Jing Xu, Junjie Yao, Deyu Cong

**Affiliations:** ^1^ School of Acupuncture and Tuina, Changchun University of Chinese Medicine, Changchun, China; ^2^ Research Center of Traditional Chinese Medicine, Affiliated Hospital of Changchun University of Chinese Medicine, Changchun, China; ^3^ Department of Science and Technology, Changchun University of Chinese Medicine, Changchun, China; ^4^ Department of Tuina, Affiliated Hospital of Changchun University of Chinese Medicine, Changchun, China

**Keywords:** extracellular matrix, type 2 diabetes, sarcopenia, collagen, integrins, skeletal muscle regeneration

## Abstract

Type 2 diabetes mellitus–related sarcopenia (T2DMRS) is a common complication in elderly and advanced diabetes patients that affects long-term prognosis and quality of life. Skeletal muscle is the main unit of glucose metabolism, and it is surrounded by extracellular matrix (ECM), which is a microenvironment that acts as an efficient highway system. The ECM is essential for cellular communication and nutrient transport and supports muscle cell growth and repair. When this “ECM highway” fails to function effectively because of damage or blockage, the development of T2DMRS can be triggered or exacerbated. In recent years, the ECM has been widely demonstrated to play a critical role in insulin resistance and skeletal muscle regeneration. However, how the remodeling of skeletal muscle ECM components specifically affects the T2DMRS mechanism of action has not been scientifically described in detail. In this review, we comprehensively summarize the T2DMRS-related mechanisms of ECM remodeling, suggesting that collagen and integrins may be potential therapeutic targets.

## 1 Introduction

Diabetes is a chronic metabolic disease that affects approximately 25% of the world’s population over 65 years of age (1). Sarcopenia is a progressive, systemic skeletal muscle disease that results in accelerated loss of muscle mass and function with age (2). Sarcopenia has been described as an emerging complication of diabetes mellitus in the elderly population, with prevalence rates ranging from 10% to 27% (3). People with type 2 diabetes mellitus (T2DM) are more likely to develop sarcopenia than those with normal blood glucose (4). T2DM and sarcopenia tend to be intertwined, and this comorbidity has become an important public health issue in today’s world.

There are complex interactions between T2DM and sarcopenia. Muscle loss in patients with T2DM is primarily associated with chronic inflammation, oxidative stress, insulin resistance, formation of advanced glycosylation end products (AGEs), and hyperglycemia, which collectively affect the muscle energy supply and protein metabolism and, consequently, the development of sarcopenia (5). Sarcopenia, in turn, has been found to be associated with an increased risk of developing T2DM (6). Skeletal muscle is the largest insulin-sensitive organ in the body. Approximately 80% of glucose in the human body is absorbed by skeletal muscle and stored in the form of glycogen (7). A decrease in the amount and function of skeletal muscle, an important tissue for peripheral glucose uptake, will diminish glucose uptake and exacerbate the development of T2DM (8). T2DM and sarcopenia are deeply intertwined pathogenetically, with skeletal muscle playing a central role in their bidirectional effects.

The extracellular matrix (ECM), an important component of the skeletal muscle niche, has been shown to be affected by aging (9) and different stages of diabetes (10, 11), and it is closely linked to the phenomenon of insulin resistance in skeletal muscle (12). However, there are fewer relevant studies available to show data on specific grading changes during diabetes onset. Therefore, more studies are needed to comprehensively assess specific markers of diabetes onset and aging. During the onset of T2DM, the ECM network in skeletal muscle undergoes dynamic changes, altering the interactions between cells and the ECM (13). Abnormal ECM remodeling disrupts insulin signaling and glucose transport (14). Therefore, the ECM plays a crucial role in regulating insulin sensitivity in skeletal muscle. Additionally, the ECM has multiple roles in the process of skeletal muscle regeneration, including providing physical support, regulating cell behavior, storing growth factors, and guiding the formation of new myofibers (11). The growth and regeneration of skeletal muscle depend on the activation of muscle-specific stem cells, known as satellite cells. The activation and differentiation of satellite cells into myoblasts and their migration, proliferation, and fusion into functional multinucleated myofibers are all related to the synthesis and degradation of ECM proteins (15). The ECM also undergoes dynamic changes during the growth or repair of skeletal muscle, experiencing extensive remodeling during muscle regeneration and continuously regulating cell proliferation, migration, and differentiation (16).

T2DM may lead to sarcopenia, and conversely, sarcopenia can initiate and exacerbate the onset and progression of T2DM; however, there is currently a notable paucity of treatment strategies for type 2 diabetes mellitus–related sarcopenia (T2DMRS). Research has demonstrated that interventions such as pharmacological treatment (17) and exercise (18) can simultaneously address diabetes and sarcopenia while remodeling the ECM. Consequently, the development of targeted therapies focusing on the ECM may represent a promising avenue for addressing this comorbidity. Given that the remodeling of skeletal muscle ECM components plays a pivotal role in enhancing insulin resistance and promoting muscle regeneration, the mechanistic implications of the ECM in the context of T2DMRS remain inadequately elucidated. We systematically collated multiple changes in the ECM in T2DM and sarcopenia, evaluated the feasibility of various key ECM constituents as potential therapeutic targets, and focused on their possible mechanisms of action in influencing T2DMRS. This review provides important theoretical foundations and offers guidance for the development of targeted ECM remodeling strategies for T2DMRS treatment.

## 2 Overview of the ECM

**Figure 1:**
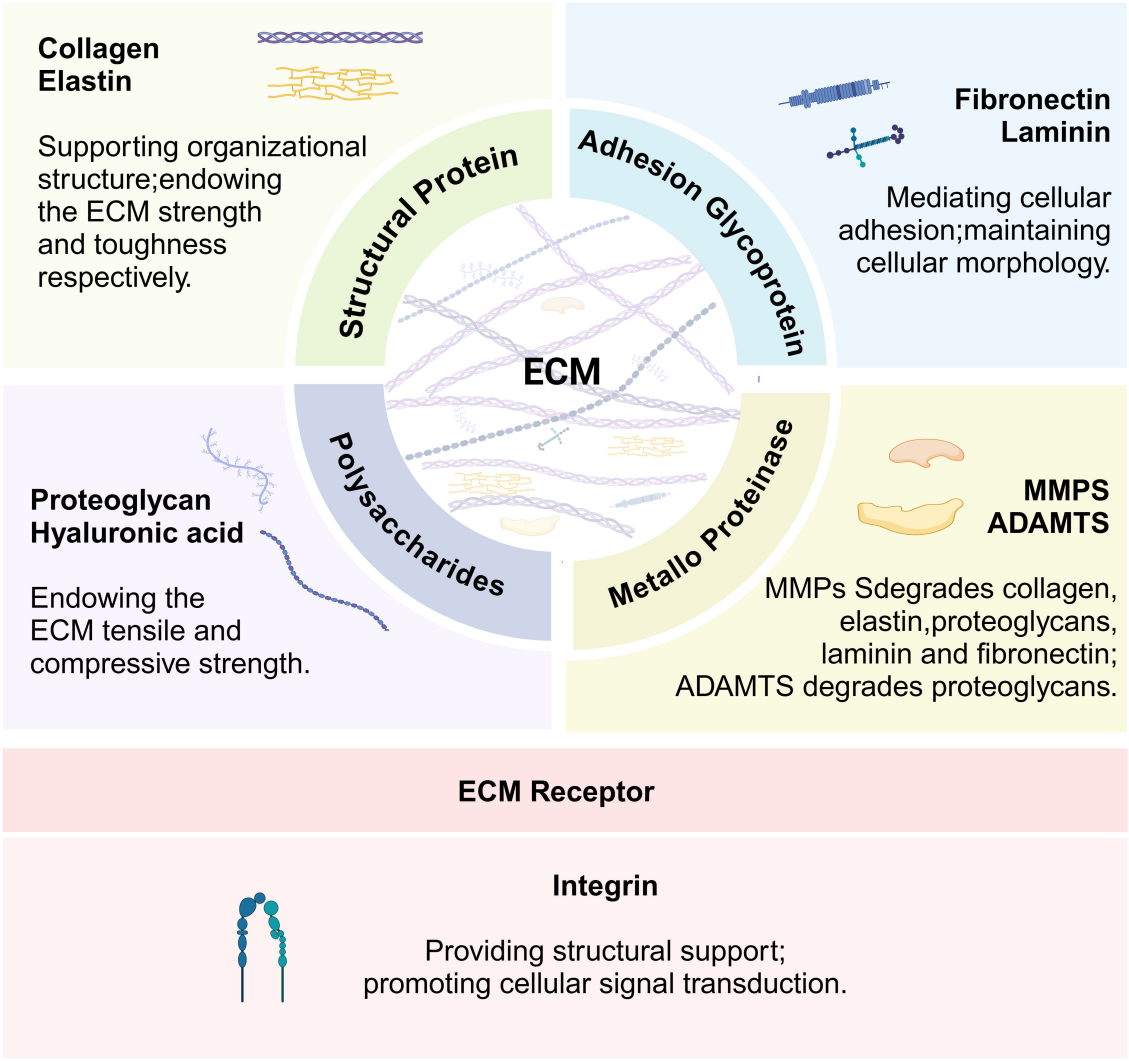
Function of key extracellular matrix (ECM) components and integrins. The figure illustrates the main functions of the key components of the extracellular matrix and their receptor integrins.

The ECM consists of more than 300 proteins, including collagen, proteoglycans, laminin, and elastin, which constitute the main elements of the ECM. Each type of protein has different physical and biochemical properties (19). As shown in **Figure 1**, structural proteins, such as collagen and elastin, give the ECM strength and toughness, respectively (20). Proteoglycans confer compressive resistance to the ECM (21), and fibronectin and laminin facilitate cell adhesion to the ECM (22). These ECM proteins provide structural support to cells and regulate cell behavior, while ECM remodeling enzymes regulate ECM turnover. The most important enzymes involved in ECM remodeling are metalloproteinases, which include two major families. The matrix metalloproteinase (MMPs) family comprises a group of proteolytic enzymes capable of degrading components such as collagen, elastin, and proteoglycans, promoting tissue remodeling and turnover (23). A disintegrin and metalloproteinase with thrombospondin motifs (ADAMTS) is also responsible for the degradation of ECM components, particularly proteoglycans (24). The ECM surrounds the cell and builds a three-dimensional network architecture. Integrins, as the main adhesion receptors of the ECM, are mediators of cell-ECM interactions and play key roles in structural support and cell signaling. The ECM receptor integrins and related proteins collectively shape the physical environment around the cells and play a critical role in structural support and cell signaling (25). ECM components are frequently remodeled by surrounding cells through degradation, synthesis, recombination, and modification and also play key roles in regulating cell viability, growth, differentiation, and metabolism (26). In skeletal muscle, each muscle fiber and its associated muscle stem cells are surrounded by the ECM (27). The vast majority of myopathies are accompanied by excessive accumulation of ECM, such as collagen deposition, which leads to muscle fibrosis, which in turn negatively affects muscle regeneration (21).

## 3 Collagen

Collagen is the most abundant structural component of the ECM. It not only supports tissues and participates in growth, differentiation, morphogenesis, and wound healing processes but also is necessary for cell adhesion and migration (28). The collagen family includes 28 different members, of which types I, III, IV, V, VI, XII, XIII, XIV, XV, XVIII, and XXII have been shown to be present in mature skeletal muscle (29). More than 90% of the collagen expressed in skeletal muscle consists of collagen types I, III, and IV.

One of the hallmarks of insulin resistance in the skeletal muscle of human and rodent populations with T2DM is collagen deposition, characterized by a marked increase in the abundance of collagen I (Col I), collagen III (Col III), and collagen IV (Col IV) (30–32). Collagen has been shown to actively drive insulin resistance (33), possibly through regulation of the insulin signaling pathway. Insulin receptor substrate 1 (IRS1) phosphorylation may be inhibited, leading to reduced phosphatidylinositol 3‐kinase (PI3K) activation and thereby affecting the phosphorylation of its downstream signaling molecule protein kinase B (Akt). Reduced Akt activation and glucose transporter type 4 (GLUT4) membrane translocation ultimately result in decreased glucose uptake capability (14). A study found that the deposition of Col III and Col IV proteins in rodents with skeletal muscle insulin resistance may be associated with the interaction of integrin α2β1 (32). Subsequently, the research team demonstrated that Col IV protein deposition is associated with reduced MMP-9 activity. In addition, COL1a1, a gene encoding the α1 chain in Col I, was observed to be associated with transforming growth factor beta (TGF-β) upregulation in T2DM rats. Given the central role of TGF-β in promoting collagen deposition and fibrosis, it is likely that COL1a1 overexpression is closely correlated with the state of inflammation and fibrosis in the diabetic setting (34). However, collagen transcription is downregulated in the skeletal muscle of streptozotocin-induced type 1 diabetes mellitus (T1DM) mice, which may be due to the inhibition of collagen synthesis by low levels of TGF-β and an accumulation of AGEs (35), and the concomitant increase in the levels of inflammatory cytokines and MMPs may also promote collagenolysis (36).

Increased collagen content is also a feature of age-related sarcopenia (37). Many aspects of collagen structure, including collagen arrangement, cross-linking, and stacking density, affect the regenerative capacity of skeletal muscle (38). Collagen deposition may be triggered by a fibrotic process possibly regulated by nuclear factor-kappa B (NF-κB) or the TGF-β/Smad signaling pathway (39). Collagen directly interacts with myosatellite cell surface receptors as a ligand, playing a key role in maintaining satellite cell homeostasis and activating repair after injury (40). In a zebrafish model of sarcopenia, an increase in collagen content paralleled a dramatic decrease in mitochondrial density between myofibers, with 100% mitochondrial damage (41). Collagen deposition causes an increase in ECM stiffness, and abnormal mechanical stresses affect cell shape as well as the mitochondrial network structure (42). As a result, excessive reactive oxygen species (ROS) are generated (43), enhancing forkhead box protein O (FOXO) transcriptional activity (44). This FOXO transcriptional activity then activates the expression of muscle atrophy F-box and muscle RING finger 1, which are muscle-specific E3 ubiquitin ligases involved in protein degradation processes that can impede muscle regeneration (45). Collagen deposition may also be involved in the inhibition of myogenic differentiation, leading to decreased expression of myogenic differentiation 1 (MyoD) and other regulators of myogenic differentiation (46).

**Figure 2:**
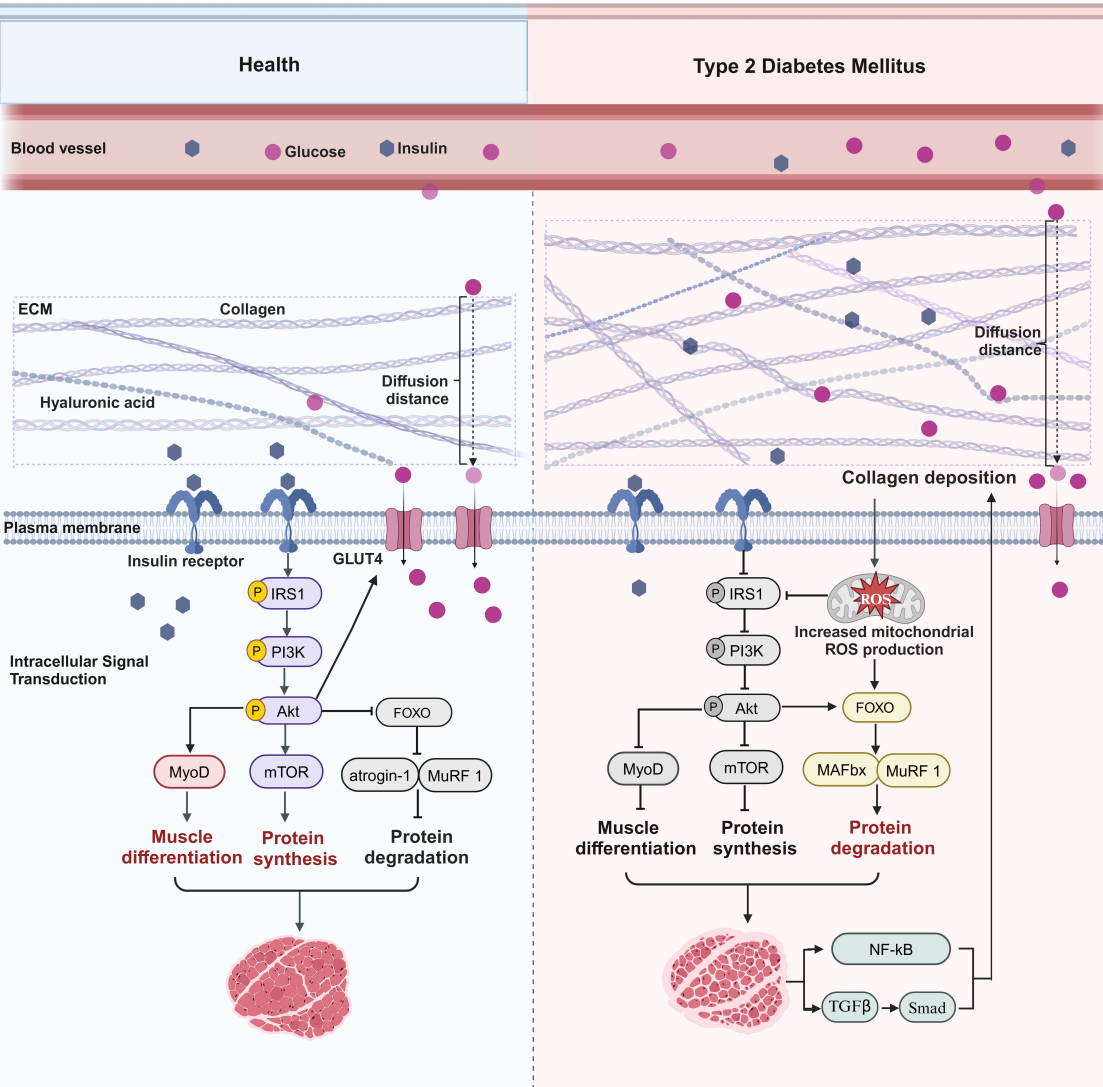
Collagen-mediated pathways in type 2 diabetes mellitus–related sarcopenia. In a healthy state, normal collagen levels allow for efficient delivery of insulin and glucose to the muscle, maintaining a healthy muscle state. Binding of insulin to its receptor and activation of insulin receptor substrate 1 (IRS1) phosphorylation subsequently recruit and activate phosphatidylinositol 3‐kinase (PI3K)/protein kinase B (Akt), which facilitates the translocation of GLUT4 to the cell membrane and increases the efficiency of glucose uptake by muscle. Akt then activates or inhibits multiple downstream targets. For instance, Akt activates the mammalian target of rapamycin (mTOR) to promote protein synthesis and inhibits forkhead box protein O (FOXO), which inhibits its downstream E3 ubiquitin ligases muscle atrophy F-box (MAFbx) and muscle RING finger 1 (MuRF-1), thereby inhibiting protein degradation. In addition, Akt promotes myogenic differentiation of satellite cells by activating myogenic differentiation regulators such as myogenic differentiation 1 (MyoD). In T2DM, collagen deposition increases the distance of the diffusion path for glucose and insulin, reduces the binding efficiency of insulin to receptors, inhibits the IRS1/PI3K/Akt pathway, and impedes the transport of GLUT4 to the cell membrane, which reduces muscle uptake and utilization of glucose. Akt subsequently activates or inhibits multiple downstream targets. mTOR is inhibited, inhibiting protein synthesis, and FOXO is activated, promoting MAFbx and MuRF-1 expression, which accelerates protein degradation. In addition, myogenic differentiation regulatory factors such as MyoD are inhibited, thereby inhibiting myogenic differentiation of satellite cells. Collagen deposition can also cause impaired mitochondrial function and increased ROS levels, which not only inhibit the insulin signaling pathway but also activate members of the FOXO family and their downstream E3 ubiquitin ligases, thereby accelerating protein degradation. Muscle loss can also cause continued collagen production through inflammatory and fibrotic pathways.

Changes in collagen content in the diabetic setting may be associated with various characteristics, such as inflammation, fibrosis, accumulation of AGEs, MMPs, integrin-mediated cell-ECM interactions, and lipid accumulation. Interestingly, the collagen content of skeletal muscle is polarized in T1DM and T2DM, and the mechanism responsible for the decrease in collagen content caused by T1DM is unclear. Combined with the body’s energy metabolism homeostasis mechanism, such a decrease may be a compensatory response to muscle tissue catabolism in the high-glycemic state. The main reason for the increase in collagen content in T2DM may be lipid accumulation. Lipid accumulation is closely related to collagen content, and lipid infusion may increase the content of collagen types I and III in human skeletal muscle (47, 48). Collagen also regulates the formation and function of adipose tissue (48). Another study supports the idea that a decrease in MMP-9 content by itself does not cause insulin resistance in skeletal muscle but does cause severe muscle insulin resistance when combined with a high-fat diet, probably because MMP-9 is not sufficient to degrade the collagen deposits caused by lipid accumulation (49). This phenomenon also provides a new perspective on why an excess supply and underutilization of lipid fuels can induce insulin resistance in skeletal muscle (30). Based on this, we speculate that the underlying mechanism by which collagen affects T2DMRS may be that collagen deposition forms a mechanical barrier that lengthens the insulin and glucose diffusion pathways, thereby impeding insulin and glucose signaling and metabolic processes in muscle and thus exacerbating skeletal muscle insulin resistance (**Figure 2**). This process may inhibit the PI3K/Akt/mTOR pathway while stimulating protein degradation through FOXO family members and their downstream E3 ubiquitin ligases and autophagy regulators (45). The imbalance between decreased muscle protein synthesis and increased degradation is an important mechanism underlying the development of sarcopenia. In addition, collagen deposition may directly contribute to the development of sarcopenia by altering the physical properties of the ECM, affecting stem cell differentiation, interfering with mitochondrial function, and promoting muscle fibrosis. It has also been found that collagen structure, rather than collagen content, is responsible for the skeletal muscle fibrosis that occurs in sarcopenia and metabolic disorders, among others (50). In conclusion, the mechanisms by which collagen accumulation and structural changes exacerbate skeletal muscle insulin resistance and muscle loss require further exploration and validation. The development of small-molecule drugs targeting collagen remodeling for the treatment of T2DMRS holds significant research value, especially for elderly patients with obesity, who are more prone to developing T2DMRS.

## 4 MMPs

MMPs are a class of protein hydrolases found in the ECM. The expression level of MMPs is significantly increased in the serum and other non-muscle tissues of patients with diabetes (51–53), a phenomenon that may be closely related to the chronic inflammation induced by diabetes and that may involve NF-κB signaling pathway activation (54). MMP-2 and MMP-9 are two important MMPs expressed in skeletal muscle (55). Their polymorphisms may influence the pathogenesis of T2DM, suggesting their potential value as biomarkers for predicting the risk and progression of T2DM (56). MMPs are mainly responsible for the degradation and turnover of matrix proteins in the ECM. When their expression is altered, it can result in the aberrant degradation of the ECM. This acts as the primary cause for the onset of chronic degenerative conditions linked to diabetes (57). Investigating MMP-mediated degradation activity in skeletal muscle through enzyme profiling, Illesca et al. discovered that the collagen deposition observed in the skeletal muscle of insulin-resistant rats could be attributed to diminished MMP-2 activity (58). Similarly, Kang et al. found that reduced MMP-9 activity also induces type III and IV collagen deposition in insulin-resistant skeletal muscle. They also reported that MMP-9 may regulate myocyte responses to insulin in mice by regulating muscle vasculature formation and potentially affecting perfusion-regulating mechanisms; therefore, another reason for the aggravation of muscular insulin resistance by MMP-9 deletion may be the reduction in capillary number (49).

Increased expression of MMP-2 and MMP-9 is seen in a variety of diseases associated with sarcopenia, and this increase may be associated with muscle fiber regeneration and tissue inflammation (59, 60). Targeting MMPs using specific strategies may prevent disease progression. Omega-3, a nutritional supplement containing EPA, DHA, and other ingredients, may reduce MMP-9 expression through macrophage-regulated mechanisms, improving myofibroblast engraftment, satellite cell activation, and muscle regeneration (61). Empagliflozin, a sodium-glucose co-transporter 2 inhibitor, may inhibit sarcopenia-induced skeletal muscle fibrosis and improve skeletal muscle function through the AMPKα/MMP-9/TGF-β1/Smad pathway (59). In addition, MMP-9 inhibition may promote myofiber regeneration by enhancing muscle contractility, improving muscle blood flow and metabolism, and modulating cell membrane signaling (62).

Tissue inhibitors of metalloproteinases (TIMPs), another class of proteins that regulate ECM synthesis and degradation, are endogenous inhibitors of MMPs in the ECM. The delicate coordination between the activity of MMPs and their inhibition by TIMPs ensures ECM homeostasis and is involved in myofibroblast migration, fusion, and a wide variety of physiological and pathological remodeling situations (63). In skeletal muscle, elevated TIMP expression inhibits MMP activity and causes collagen accumulation and cross-linking (64). The MMP/TIMP ratio usually determines the extent of ECM protein degradation and tissue remodeling (65). Dysregulation of the MMP-2/TIMP-2 and MMP-9/TIMP-1 balance may directly cause collagen disorders in patients with diabetes, and lowering the ratio may attenuate collagen deposition (66). A skeletal muscle MMP-1/TIMP-1 imbalance during the aging process also increases the number of collagen fibers, which in turn affects skeletal muscle mass and function and leads to sarcopenia (67).

Currently, the MMPs that have garnered extensive research attention in the realms of T2DM and sarcopenic diseases are primarily MMP-2 and MMP-9. Interestingly, their changes during disease progression exhibit opposing trends, which seemingly complicates the role of MMPs in T2DMRS. However, integrative analysis has revealed that the inflammatory responses and collagen deposition associated with T2DMRS may increase MMP expression levels. Consequently, MMP expression in T2DMRS may be elevated, and yet, the degradation activity of these MMPs in insulin-resistant skeletal muscle may be diminished. This presents a scenario where, despite increased expression, the MMPs remain insufficient to effectively degrade the deposited collagen. This suggests that MMP activity, rather than quantity, is more significant in collagen degradation. Therefore, understanding the relationship between MMP quantity and activity throughout disease progression is a crucial avenue for future research. Additionally, as TIMPs are MMP antagonists, their expression levels indirectly influence collagen alterations. Therefore, the dynamic balance between MMPs and TIMPs may be pivotal in maintaining collagen homeostasis. Clarifying the patterns of change in MMP activity and TIMP inhibitory effects could contribute to the development of therapeutic strategies for T2DMRS.

## 5 Hyaluronic acid

Hyaluronic acid (HA) accumulation is involved in the pathogenesis of diabetes (68). HA synthesis may be caused by the upregulation of NF-κB–controlled hyaluronan synthase 2 and is coordinated with the synthesis of chemical elicitors of HA cross-linking (69). HA increases the residual collagen content in diabetic rats (70); this implies that HA may be indirectly involved in the development of diabetes by affecting ECM remodeling. Kang et al. found for the first time that the HA content is increased in the ECM of insulin-resistant skeletal muscle and that the intravenous injection of long-acting pegylated human recombinant PH20 hyaluronidase can reverse muscle insulin resistance by decreasing HA levels in the muscle ECM (71). HA also regulates adipose tissue function and influences adipogenesis (72); therefore, HA has value as a research topic in studies of T2DMRS combined with obesity.

The expression level of cluster of differentiation 44 (CD44), a major HA receptor, is positively associated with T2DM, and it influences insulin resistance processes in skeletal muscle (73). HA-mediated insulin resistance in skeletal muscle does require the involvement of the CD44 receptor, and CD44-knockout mice show improved insulin resistance in skeletal muscle along with enhanced muscle vascularization, suggesting that HA-CD44 signaling may be involved in the pathogenesis of insulin resistance and T2DM by modulating the transport of insulin and glucose in muscle (71). As a candidate gene for the development of obesity and diabetes, CD44 may be a key mediator of the systemic inflammation response associated with obesity and diabetes, participating in the regulation of inflammatory responses. Anti-CD44 antibody therapy has been shown to possibly lower blood glucose levels while inhibiting macrophage accumulation (74). However, the influence of HA and its receptors on skeletal muscle regeneration remains unexplained. HA and its receptors may improve T2DMRS via a mechanism that addresses insulin resistance: by allowing glucose to effectively enter muscle tissue, HA and its receptors may ensure an adequate supply of nutrients to the muscle, subsequently ameliorating sarcopenia. Additionally, given its effect on adipose tissue, the HA-CD44 pathway may emerge as a novel target for treating T2DMRS in conjunction with obesity.

## 6 Laminin

Laminin is a heterotrimeric structural protein in the basal lamina of skeletal muscle fibers that surrounds the myofibers and forms an ecological niche for stem cells, providing an important scaffold for tissue development, maintenance, and function (75). The biological functions of laminin are largely realized through interactions with specific cell surface receptors, such as integrin family members. Specifically, satellite cells and myofibroblasts interact with laminin through integrin α7β1. This interaction plays a critical role in several key processes in satellite cells, including proliferation, adhesion, migration, and, ultimately, differentiation (76). For example, laminin-211, by binding to integrin α7β1, may promote the activation of key cell survival signaling pathways, such as PI3K/Akt (77). There are no studies revealing the expression of laminin in sarcopenia, but it has been found that treatments targeting laminin-111 may also promote the value-added activation of satellite cells by repairing the integrin microenvironment, resulting in effective muscle regeneration (78). In addition, laminin-111 may play a role in mechanical stability. It reinforces muscle segments to increase their resistance to the shear stresses generated during muscle contraction, thus stabilizing muscle segments and effectively protecting muscle tissues from damage (79). Research has also revealed that the utilization of laminin-111 in the development of biomaterial scaffolds may enhance satellite cell activity and suppress the deposition of fibrotic tissue, thereby enhancing muscle fiber regeneration (80).

The specific role of skeletal muscle laminin in improving T2DM has not been supported by evidence. However, it has now been found that laminin and its receptor may improve the survival and function of human pancreatic islets (81), and hydrogels containing laminin have also been investigated as materials for the delivery of insulin-secreting tissues (82). Therefore, laminin may also serve as a potential therapeutic target for sarcopenia associated with T2DM, operating primarily through its interactions with integrin receptors to activate key signaling pathways that support the proliferation, migration, and differentiation of muscle satellite cells. This activity facilitates muscle regeneration and enhances muscle mass, thereby improving glucose uptake and utilization, alleviating insulin resistance, and fostering a positive feedback loop that hampers disease progression.

## 7 Fibronectin

Fibronectin is the major non-collagenous glycoprotein in the ECM and basement membrane and plays a central role in cell adhesion by regulating cell polarity, differentiation, and growth. The effects of diabetes on fibronectin expression in skeletal muscle have not been fully explored, but it has been found that patients with T2DM exhibit fibronectin accumulation in the liver (83), kidneys (84), and other tissues. This fibronectin accumulation may involve the activation of TGF-β, MAP kinases, and connective tissue growth factors. Fibronectin released from skeletal muscle may mediate exercise-induced communication between muscle and the liver through hepatic integrin α5β1 and its downstream pathways, facilitating systemic autophagy activation and enhancing overall insulin sensitivity (83). The interactions between fibronectin and integrins may also trigger biomechanical regulatory mechanisms in which the binding state of fibronectin to integrin receptors changes according to mechanical stress, which in turn affects the behavior of tip cells and promotes or inhibits vascular growth and branching, with important implications for muscle blood supply and nutrient transport (85). Therefore, the interactions between fibronectin and integrins deserve attention. These interactions not only provide a physical connection between the cell and the surrounding ECM but also may influence cell migration, proliferation, and differentiation by converting mechanical stimuli into intracellular signals through integrin mechanical switches.

Fibronectin is the preferred adhesion substrate for muscle stem cells. In aging skeletal muscle, decreased levels of fibronectin, which regulates the p38 mitogen-activated protein kinase (p38) and extracellular signal–regulated protein kinase (ERK) senescence pathways via integrin β1 and focal adhesion kinase (FAK), impair the functioning of muscle stem cells and negatively affect the regenerative capacity of skeletal muscle (86). Therefore, targeting and elevating fibronectin levels may open new pathways for T2DMRS treatment. Additionally, research has indirectly corroborated the significance of increased fibronectin levels in muscle regeneration. The transient accumulation of fibronectin in muscle after injury is vital for the activation and proliferation of satellite cells, which are muscle stem cells. The underlying mechanism may involve the interaction between fibronectin and the frizzled-7/syndecan-4 receptor complex, which activates the Wnt7a signaling pathway, subsequently inducing the expansion of the satellite cell pool (87). However, when fibronectin accumulates excessively over time, fibrotic lesions can develop in skeletal muscle (88), hindering the function of satellite cells and adversely affecting the normal repair processes of muscle tissue. Hence, we speculate that a moderate upregulation of fibronectin expression is capable of activating the positive progression of muscle regeneration. Insufficient or excessive upregulation of fibronectin expression may have adverse effects on the regenerative capacity of muscle. Thus, the adaptive elasticity of fibronectin expression across different stages of T2DMRS is a pressing subject warranting further exploration.

## 8 Fibromodulin

Fibromodulin (FMOD) is a small leucine-rich proteoglycan in the skeletal muscle ECM with functions including the promotion of migration and angiogenesis. FMOD also has anti-inflammatory, antifibrotic, and repair properties. Skeletal muscle development and repair are highly regulated by FMOD (89). FMOD expression is highly upregulated during muscle generation, which may be attributed to the ability of FMOD to enhance the recruitment of MSCs to areas of muscle damage or atrophy. Further studies have confirmed that knocking down the FMOD gene in a mouse model significantly reduces the number of myotubes formed and suppresses the expression of genes related to muscle differentiation (90). In addition, FMOD can regulate collagen cross-linking, stacking, assembly, and fiber structure through multivalent interactions (89). It regulates the activity of muscle growth inhibitor (MSTN) in the collagen matrix by modulating COL1a1 expression. The affinity between FMOD and MSTN determines the binding efficiency of MSTN to the receptor activin receptor type IIB, a process that directly affects the activity of the TGF-β/Smad signaling pathway, which then regulates cell growth and differentiation (90). FMOD may also facilitate the influx of calcium ions by activating calcium channels in myoblasts, thereby stimulating their differentiation into myotubes (91). Furthermore, FMOD is effective in protecting myoblasts from the detrimental effects of excessive lipid accumulation (92), which is beneficial for muscle regeneration.

Research on FMOD and diabetes indicates that FMOD can downregulate genes related to diabetes, counteracting the effects of aging (92). FMOD can also suppress TGF-β1 levels in rat models of streptozotocin-induced diabetes (93). These findings suggest the potential role of FMOD in T2DMSR treatment approaches. However, there is currently a lack of direct and robust experimental evidence. Further validation is needed to confirm the inhibitory effect of FMOD on the TGF/Smad signaling pathway and clarify the mechanisms that reduce skeletal muscle fibrosis and promote muscle regeneration.

## 9 Decorin

Decorin is an important component of the ECM and belongs to a family of small leucine-rich proteoglycans. It not only provides structural support to cells, but also regulates cell behavior and function, and is involved in cell signaling and regulation. Myostatin, as a member of the TGF-β superfamily of growth factors, is an important negative regulator of skeletal muscle mass. Decorin is able to bind to and block the effects of myostatin (94). TGF-β1 stimulates muscle growth inhibitor expression, whereas decorin binds to TGF-β1 and blocks its signaling, thereby ameliorating muscle fibrosis (95–97). In addition, decorin has the potential to enhance the proliferation and differentiation of C2C12 myofibroblasts by inhibiting myostatin activity, thereby promoting muscle regeneration (98). Studies have shown that exercise may lead to elevated decorin levels and downregulate myostatin through competitive binding to promote myofiber growth (99). Thus, the interaction between decorin and myostatin may be critical. In addition, decorin secreted by skeletal muscle might protect human islets from inflammation-induced cell death in T2DM patients, thereby restoring pancreatic function and reversing T2DM-related gene expression. Thus, targeting upregulation of decorin may be potentially beneficial for T2DMSR therapy, and muscle growth inhibitors may also play an important role in this process of conversion.

To sum up, **Table 1** summarizes the remodeling of these major skeletal muscle extracellular matrix components in patients and animal models of type 2 diabetes and sarcopenia.

**Table 1:** Remodeling of major components of the skeletal muscle extracellular matrix in type 2 diabetes mellitus and sarcopenia.

Skeletal muscle Extracellular matrix	Type 2 diabetes mellitus	Sarcopenia
Collagen I	↑[human (30, 31)]	↑[C57BL/6J mice (37)]
Collagen III	↑[human (30, 31)]	↑[C57BL/6J mice (37)]
Collagen IV	↑[C57BL/6J mice (32)]	—
Matrix metalloproteinase-2	↓[C57BL/6J mice (58)]	↑[C57BL/6J mice (60)]
Matrix metalloproteinase-9	↓[C57BL/6J mice (49)]	↑[C57BL/6J mice (59)]
Hyaluronic acid	↑[C57BL/6J mice (71)]	—
Fibronectin	—	↓[C57BL/6J mice (88)]

## 10 Integrins

Integrins are transmembrane proteins located on the cell surface that serve as bidirectional links between the ECM and the cellular cytoskeleton. They transmit external stimuli to regulate cellular processes, mediating the conversion of mechanical forces into chemical signals and enabling biomechanical signal transduction through force-chemical coupling (100). An association between integrins (101) and the onset of diabetes has been noted, with integrins even participating in the regulation of insulin activity in muscle during the early stages of insulin resistance (102). Seven α subunits of integrins are expressed in skeletal muscle: α1, α3, α4, α5, α6, α7, and αV, all of which are associated with the β1 subunit of integrins (12). The integrin β1 receptor is associated with the actin cytoskeleton via talin, and integrin β1–deficient mouse skeletal muscle shows reduced levels of talin and F actin, reduced AktSer-473 phosphorylation, and significantly reduced integrin-linked kinase (ILK) expression, which may contribute to the impairment of insulin-stimulated glucose uptake and glycogen synthesis in skeletal muscle (103). Research has found that the aggregation of integrin β1 may facilitate the phosphorylation of the insulin receptor and IRS1, thereby enhancing PI3K activity and stimulating the activation of Akt/PKB (104). Integrins may also regulate signaling pathways for collagen synthesis, contributing to the maintenance of ECM homeostasis. Integrin α1β1 is antifibrotic and negatively regulates collagen production, whereas integrin α2β1 is pro-fibrotic, increases ROS production, and positively regulates the synthesis of collagen (e.g., collagen IV) (105). Integrin-collagen interactions affect skeletal muscle insulin and glucose metabolism. Integrins may also affect vascular density and function. The functions of integrins α1β1 and α2β1 in endothelial cells are antagonistic. The expression of α1β1 promotes vascular network formation (106), whereas α2β1 restricts vascular growth (107). It is evident that integrins may be involved in regulating the homeostasis of the vascular system under diabetic conditions.

Integrin β1 maintains skeletal muscle homeostasis and sustains the expansion and self-renewal of this stem cell pool during regeneration (108). Defective integrin signaling affects fibroblast growth factor responsiveness, which further contributes to impaired satellite cell proliferation and muscle regeneration (109). Activation of integrin β1 signaling has the potential to restore the sensitivity of fibroblast growth factor in aged skeletal muscle and enhance muscle regeneration, which may involve the activation of their common downstream effectors, ERK and Akt (108). Fibroblast growth factor receptor binding to fibroblast growth factor activates a number of intracellular signaling pathways, including p38α/β MAPK, ERK MAPK, PI3K/Akt, phospholipase C gamma/protein kinase C, and signal transducer and activator of transcription signaling, which may regulate satellite cell function to promote skeletal muscle regeneration (110). Integrin α7β1 is the major laminin receptor on adult skeletal myoblasts and adult muscle fibers that connects laminin to the cytoskeleton of the cell, and targeted deletion of the integrin α7 subunit gene results in altered expression of the laminin-α2 chain, resulting in muscle loss (111). In addition, integrin β3 has been shown to play an important role in muscle regeneration, and it may inhibit TGF-β1/Smad signaling by regulating macrophage infiltration and polarization, thereby reducing muscle fibrosis and promoting muscle regeneration (112).

Integrins, as pivotal mechanical sensors and regulators of growth induced by mechanical loading, have been shown to modulate muscle cell function in response to exercise and similar modalities (113). The expression of integrin α7 increases with exercise. Following repeated centrifugation exercise, integrin α7β1 can activate the Akt/mTOR/p70S6K signaling pathway, thereby leading to efficient muscle growth induced by exercise (114). Massage intervention after prolonged overloading exercise can enhance the expression of membrane proteins integrin β1 and basement membrane laminin 2, thus increasing muscle strength and promoting skeletal muscle repair (115). Following eccentric bicycle training, the increase in the level of the integrin β1-ILK-RICTOR-Akt complex protein in human muscle leads to a corresponding increase in muscle mass and strength (116).

ILK is an important downstream effector of integrins in skeletal muscle that connects integrins to the actin cytoskeleton and to many signaling pathways involved in integrin (117). ILK is involved in the regulation of a wide range of cellular biological functions, including cell differentiation, proliferation, migration, and apoptosis, through activation in response to integrins (118). ILK is absent in skeletal muscle. The muscle atrophy that occurs with ILK deficiency in skeletal muscle closely resembles the phenotype of integrin α7β1–deficient mice (119). Upregulation of the integrin α7-ILK-Akt signaling pathway represents an important compensatory mechanism that stabilizes and repairs myofibrillar architecture in response to muscle injury (116). mTOR is a key regulator of protein synthesis and myofiber growth and has two distinct core components of multiprotein complexes, namely mTOR complex 1 (mTORC1) and mTOR complex 2 (mTORC2) (120). mTORC1 is the primary regulator of protein synthesis (121), while mTORC2 is a major regulator of cytoskeletal structure and cell survival (122). Integrin α7β1 promotes myofibril growth via FAK/mTORC1, which is the classical integrin-mediated pathway emphasized in the current literature. In contrast, Boppart et al. suggested that integrins may not rely on the role of mTORC1 in muscle growth after mechanical stimulation but rather sustain the maintenance and remodeling of muscle architecture through an integrin-ILK-mTORC2-YAP–driven mechanosensing mechanism (113). During the skeletal muscle response to mechanical stress, the β1 integrin-ILK complex plays a key role by facilitating insulin-like growth factor 1 receptor and IRS signaling to the PKB/Akt signaling pathway (123).

FAK also plays a key role in integrin signaling. A decreased regenerative capacity of skeletal muscle stem cells is associated with impaired FAK signaling during aging (86). Liang et al. analyzed miRNA profiles in the skeletal muscle of aged rats after exercise intervention and found that Fak may be a hub gene associated with aging-induced muscle loss (124). Luo et al. found that phosphorylation of FAK by integrin β1 may activate downstream signaling pathways, such as ERK and PI3K/Akt, and promote muscle regeneration in differentiated muscles (125). Furthermore, in skeletal muscle cells, FAK promotes normal insulin-stimulated glucose transport and glycogen synthesis by maintaining the integrity of the actin cytoskeleton (126). Bisht et al. observed that insulin resistance led to reduced tyrosine phosphorylation of FAK in C2C12 cells. They found that FAK may stimulate GLUT4 translocation by positively regulating the IRS1/PI3K/PKC signaling pathway, thereby improving insulin sensitivity and glucose uptake (127). Subsequently, in a FAK-silenced mouse model, they found significantly reduced levels of IRS1 and Akt-Ser473 phosphorylation in muscle and the liver, which further contribute to insulin resistance (128).

**Figure 3:**
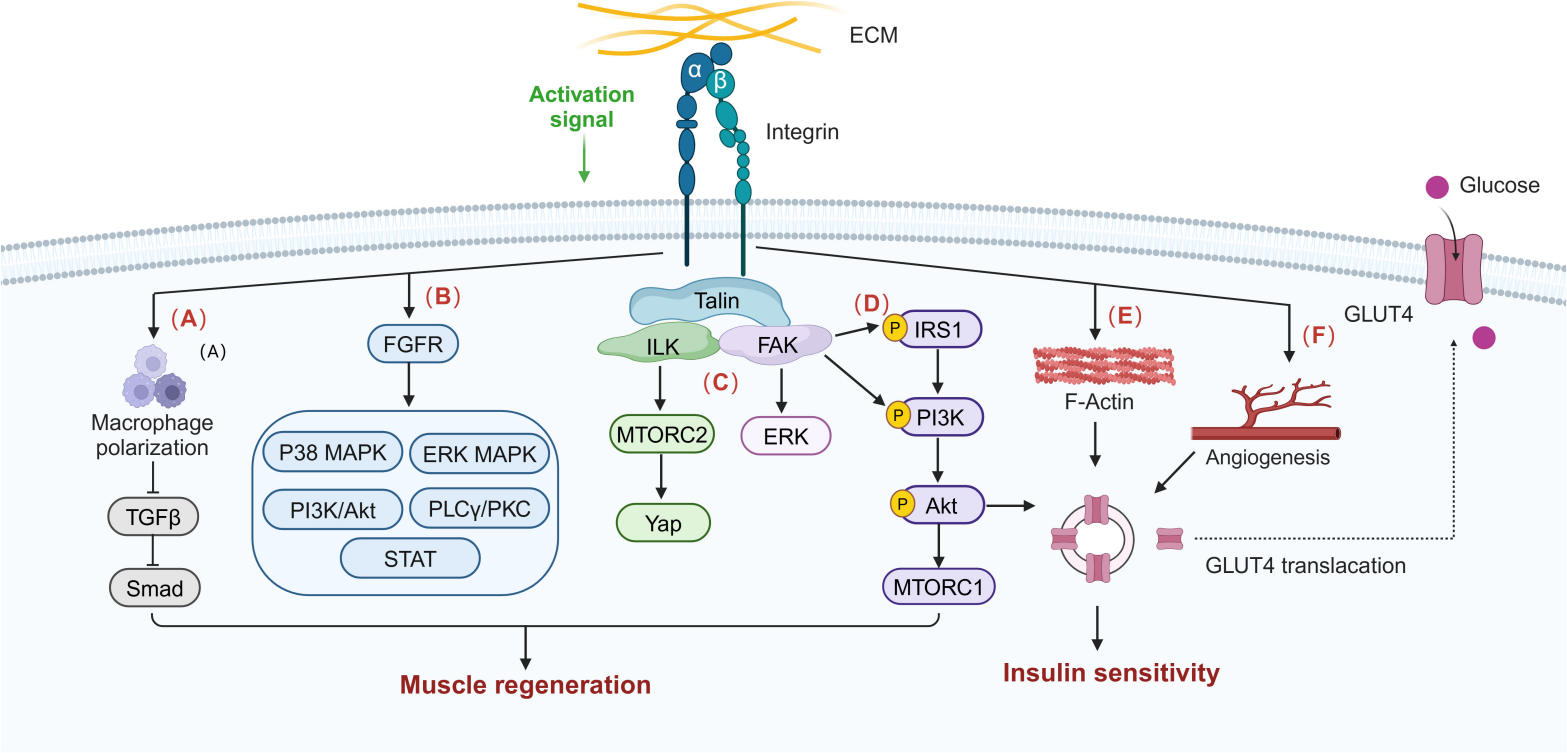
Integrin signaling influences potential pathways in skeletal muscle insulin sensitivity and muscle regeneration. The normal activation of integrin signaling regulates macrophage polarization and infiltration and inhibits macrophage transforming growth factor beta (TGF-β)/Smad signaling, thereby improving muscle fibrosis and promoting muscle regeneration. **(A)** Integrin can activate skeletal muscle regeneration and repair signal pathways such as p38 mitogen-activated protein kinase (MAPK), extracellular signal–regulated kinase (ERK) MAPK, PI3K/Akt, phospholipase C gamma (PLCγ)/protein kinase C (PKC), and signal transducers and activators of transcription (STAT) through interaction with fibroblast growth factor receptor (FGFR). **(B)** Integrins also activate the ILK/MTORC2/Yap, FAK/ERK, and FAK/IRS1/PI3K/Akt/MTORC1 pathways to synergistically promote skeletal muscle regeneration. **(C)** Integrins are involved in maintaining the stability of the actin cytoskeleton, **(E)** promoting insulin signaling pathway activation and **(D)** muscle capillary neovascularization, and **(F)** thereby stimulating GLUT4 translocation and increasing glucose uptake and insulin sensitivity.

Integrins and their related downstream targets may regulate skeletal muscle insulin resistance in T2DMRS in several ways. First, integrin-related proteins increase cytoskeletal stability and may stimulate glucose uptake and glycogen synthesis processes in skeletal muscle by facilitating actin remodeling. Second, integrins and their downstream proteins may enhance the effective transport of insulin and nutrients within muscle tissue by promoting muscle angiogenesis. Third, integrin proteins can sense changes in ECM mechanical properties and mediate the conversion of mechanical forces to chemical signals, which may help to maintain normal insulin signaling and muscle structure remodeling to ameliorate skeletal muscle insulin resistance. Integrins may promote skeletal muscle regeneration through mechanisms such as activation of muscle stem cells, maintenance of muscle architecture, modulation of inflammatory responses, inhibition of fibrosis, and mediation of mechanical signaling for activation of key growth pathways, which in turn improves muscle insulin resistance. There are many members of the integrin family, some of which are even antagonistic to each other. Integrin β1, especially α7β1 in skeletal muscle, has been demonstrated to play a positive regulatory role in diabetes mellitus and sarcopenia. In addition, mechanotherapy, which can activate integrin sensors and promote force-chemical signaling, is a therapeutic strategy that deserves in-depth exploration. In summary, integrins and related proteins have a compounding role in regulating insulin sensitivity and muscle regenerative capacity in skeletal muscle, as shown in **Figure 3**, which makes them highly promising targets when exploring therapeutic options for T2DMRS.

## 11 Therapeutic strategies

**Figure 4:**
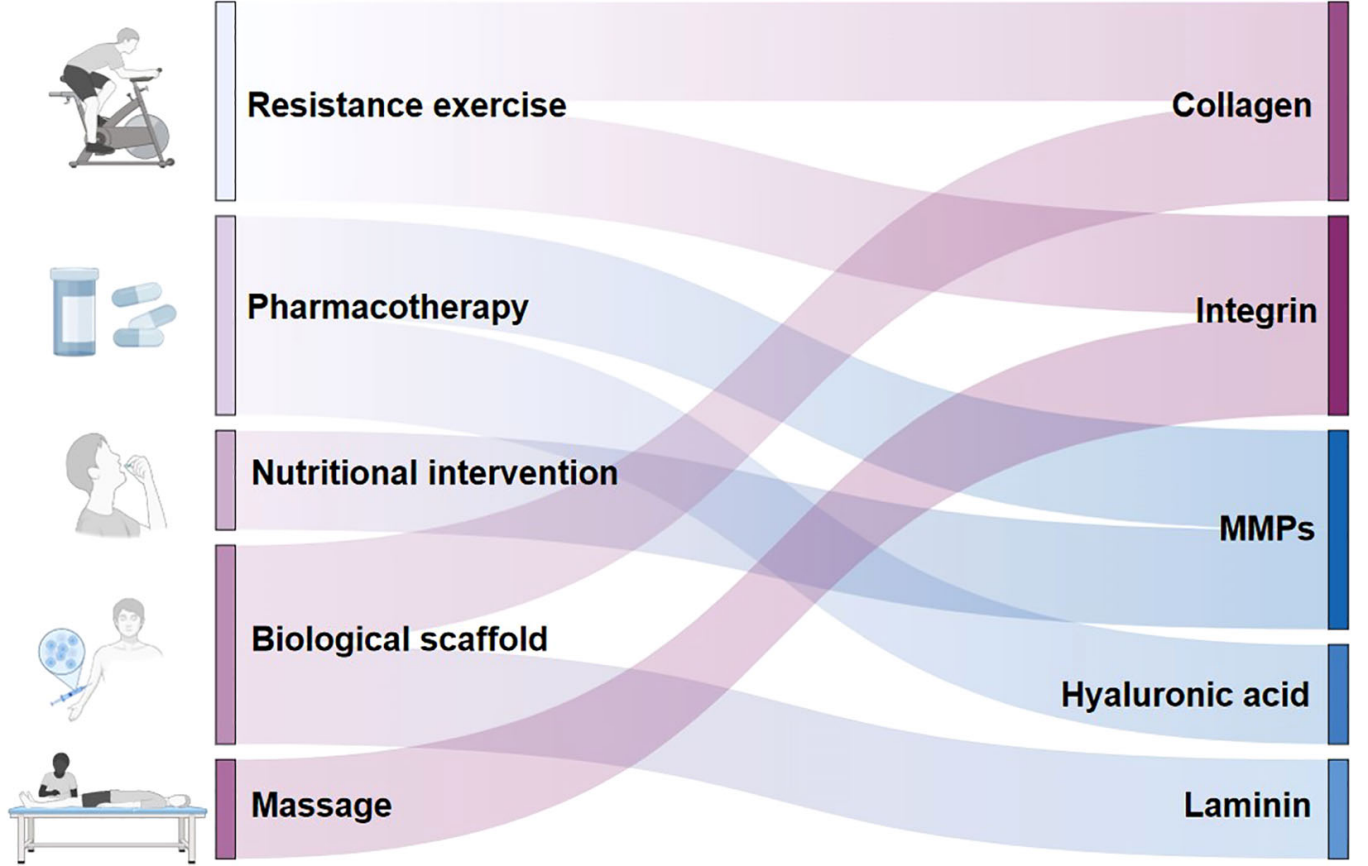
Potential extracellular matrix-targeted therapies for type 2 diabetes mellitus–related sarcopenia. This figure illustrates potential ECM targeted therapies for the treatment for T2DMRS. It also distinguishes the ECM remodeling targets of different therapies.

There is no recognized specific treatment for T2DMRS. Because of the multiple bidirectional effects between sarcopenia and T2DM, there is some commonality between the two in terms of treatment strategies. Currently, some therapies remodel the ECM while treating diabetes or sarcopenia. Therefore, targeting the ECM may be an effective strategy for treating T2DMRS. The development of targeted therapies focusing on the ECM is expected to be an important way to address this complication. As shown in **Figure 4**, studies have found that exercise training remodels collagen (18), and integrins (110, 114); drugs modulate collagen (129), hyaluronic acid (71), and MMPs (59); nutritional interventions also have an effect on MMPs (61); and massage may modulate integrin signaling (115). In addition, researchers have attempted to develop a variety of biomaterials and scaffolds that mimic skeletal muscle ECM components such as collagen (130) and laminin (82).

## 12 Conclusions and perspectives

The ECM is critical for maintaining homeostasis in skeletal muscle. In diabetes mellitus and sarcopenia, the ECM in skeletal muscle undergoes extensive remodeling, which involves an abnormal accumulation of collagen, HA, and other components. The resulting ECM highway blockage forms a physical barrier that prevents the efficient transport of nutrients such as insulin and glucose to the muscle. This may be one of the mechanisms underlying T2DMRS development. In addition, abnormal ECM remodeling can damage the highway to the muscle, which may inhibit insulin signaling, impair the normal function of insulin, affect mechanical signaling in skeletal muscle, and affect muscle regeneration. Therefore, unclogging and repairing this hidden highway may be an effective strategy for the treatment of T2DMRS. Targeting the ECM may promote muscle regeneration by increasing skeletal muscle insulin sensitivity and improve T2DM insulin resistance by increasing muscle volume, improving glucose uptake and utilization, and subsequently producing a virtuous circle that counteracts disease development.

The key to targeting ECM remodeling as a T2DMRS treatment approach lies in restoring the dynamic balance of the ECM. After comprehensively analyzing the current literature, we have identified a striking common feature: collagen deposition is prevalent in patients with T2DM and sarcopenia. Furthermore, inhibition of collagen synthesis or promotion of its degradation ameliorates both skeletal muscle insulin resistance and sarcopenia, which makes collagen a promising target in the development of therapeutic strategies for T2DMRS. MMPs, TIMPs, and integrins associated with the dynamic homeostasis of collagen also have significant research value. Other ECM components and related proteins also have some potential as targets for disease intervention, but more research is needed.

Correcting sarcopenia is expected to be the cornerstone of improving glycemic stability and long-term prognosis in elderly patients with T2DM. The development of novel drugs or therapies targeting the ECM is expected not only to lower blood glucose levels but also to promote muscle health, thereby significantly improving clinical benefits and increasing the healthy life expectancy of elderly patients with diabetes. However, a number of potential challenges remain in this process. First, ECM components are complex and diverse and vary across tissues and disease states, which makes it difficult to develop drugs or other therapies that can specifically target skeletal muscle ECM components. Therefore, we need to strengthen basic research on ECM components in different tissues and disease states, such as using histological techniques to identify altered components specific to the T2DMRS state, or consider a multi-targeted drug development strategy. In addition, the dense structure of the ECM and its role as a physical barrier may limit the effective delivery of therapeutic drugs. This could be improved by developing novel drug delivery vehicles, such as nanotechnology carriers, optimizing topical delivery modalities, and exploring combined applications with physical therapies to enhance drug penetration. It should not be overlooked that adverse effects associated with muscle fibrosis may be triggered when modulating ECM remodeling. For example, drugs that down-regulate MMP activity by targeting it may lead to a decrease in collagen degradation capacity, thus causing muscle fibrosis. Therefore, in future studies, the issue of precise dosage needs to be explored in conjunction with the use of antifibrotic adjuvants to mitigate side effects. Despite the many challenges of targeted ECM therapy, it is expected to bring new breakthroughs in the treatment of T2DMRS through in-depth study of its structure and function, innovative technological tools, and optimization of combined treatment protocols.

As research has progressed, our understanding of the role of the ECM has evolved from the traditional perception of it being a structural scaffold to that of it serving as a key signaling molecule that regulates integrin-mediated mechanotransduction pathways. Mechanical stimulation has been shown to promote ECM remodeling and integrin signaling in skeletal muscle. The conversion from mechanical stimulation to chemical signals regulates insulin resistance and muscle regeneration in skeletal muscle. Exercise is currently the preferred strategy for sarcopenia treatment, but its mechanism of regulating the ECM and integrins needs to be further elucidated to provide a scientific basis for the continued development of evidence-based exercise programs targeting the ECM. Considering the motor impairment and related risks faced by elderly patients with T2DM, non-pharmacological therapies that are suitable for patients with limited movement, such as massage and acupuncture, have broad application prospects. In summary, the hidden highway comprising the ECM and integrins may become a risk prediction marker and potential target for T2DMRS treatment.
